# Supplementary material for: Splice-Junction-Based Mapping of Alternative Isoforms in the Human Proteome
Source: Cell Rep. Author manuscript; Available in PMC 2020 Jan 15. (PMC6961840; doi:10.1016/j.celrep.2019.11.026)
Supplement: 2 [file NIHMS1546469-supplement-2.zip › DF1/PXD010154/Thyroid/Thyroid_11_PPP1R12A_ISEMEEELKR.pdf]

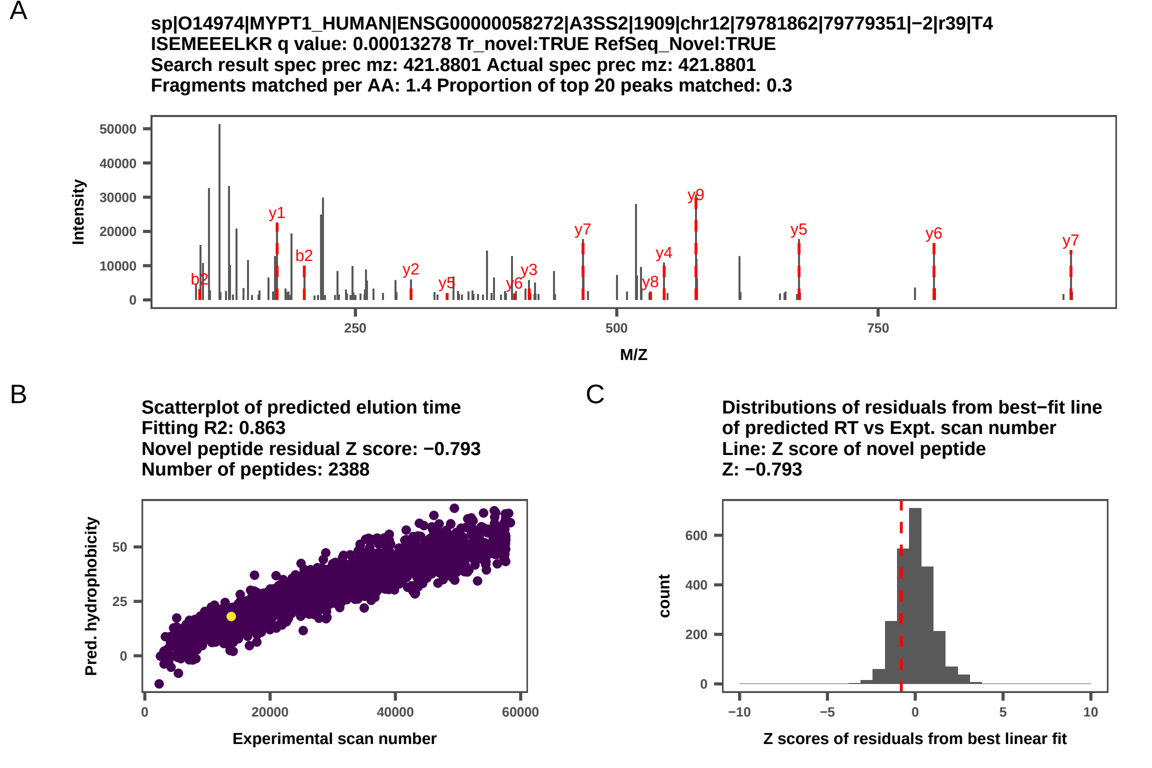
A
sp|O14974|MYPT1_HUMAN|ENSG00000058272|A3SS2|1909|chr12|79781862|79779351|−2|r39|T4
ISEMEEELKR q value: 0.00013278 Tr_novel:TRUE RefSeq_Novel:TRUE
Search result spec prec mz: 421.8801 Actual spec prec mz: 421.8801
Fragments matched per AA: 1.4 Proportion of top 20 peaks matched: 0.3
Intensity
M/Z
b2
y1
b2
y2
y5
y6
y3
y7
y8
y4
y9
y5
y6
y7
B
Scatterplot of predicted elution time
Fitting R2: 0.863
Novel peptide residual Z score: −0.793
Number of peptides: 2388
Pred. hydrophobicity
Experimental scan number
C
Distributions of residuals from best−fit line
of predicted RT vs Expt. scan number
Line: Z score of novel peptide
Z: −0.793
count
Z scores of residuals from best linear fit
